# Analysis of the benefit of sequential cranial radiotherapy in patients with EGFR mutant non-small cell lung cancer and brain metastasis

**DOI:** 10.1007/s12032-016-0811-3

**Published:** 2016-07-22

**Authors:** Seonggyu Byeon, Jun Soo Ham, Jong-Mu Sun, Se-Hoon Lee, Jin Seok Ahn, Keunchil Park, Myung-Ju Ahn

**Affiliations:** 1 Department of Medicine, Samsung Medical Center, Sungkyunkwan University School of Medicine, Seoul, Korea; 2 Division of Hematology-Oncology, Department of Medicine, Samsung Medical Center, Sungkyunkwan University School of Medicine, 81 Irwon-ro, Gangnam-gu, Seoul, 135-710 Korea

**Keywords:** Non-small cell lung cancer (NSCLC), Brain metastasis, Epidermal growth factor receptor (EGFR), Tyrosine kinase inhibitor (TKI), Whole brain radiation therapy (WBRT), Stereotactic radiosurgery (SRS)

## Abstract

Although cranial radiotherapy is considered the standard treatment for brain metastasis (BM), EGFR tyrosine kinase inhibitors (TKIs) have shown promising activity in EGFR mutant non-small cell lung cancer (NSCLC) patients with BM. However, the efficacy of sequential cranial radiotherapy in patients with EGFR mutant NSCLC who are treated with EGFR TKIs remains to be determined. Patients with NSCLC who harbored an EGFR mutation and whose BM had been treated with EGFR TKIs were retrospectively reviewed. The clinical outcomes of patients treated with EGFR TKIs alone and those treated with cranial radiotherapy followed by EGFR TKIs (additive therapy) were compared. Of the 573 patients with NSCLC with BM who harbored an EGFR mutation and had received EGFR TKIs, 121 (21.1 %) had BM at the time of initial diagnosis. Fifty-nine (49 %) patients were treated with additive therapy, whereas 62 (51 %) patients were treated only with EGFR TKIs. No significant differences were observed between the additive therapy group and the EGFR TKI alone group regarding intracranial progression-free survival (PFS) (16.6 vs 21.0 months, *p* = 0.492) or extracranial PFS (12.9 vs 15.0 months, *p* = 0.770). The 3-year survival rates were similar in both groups (71.9 vs 68.2 %, *p* = 0.675). Additive therapy consisting of cranial radiotherapy followed by EGFR TKI treatment did not improve OS or intracranial PFS compared with EGFR TKI treatment alone in EGFR mutant NSCLC patients with BM. Further prospective studies are needed to determine the precise benefits of sequential cranial radiotherapy in EGFR mutant NSCLC treated with EGFR TKIs.

**Electronic supplementary material:**

The online version of this article (doi:10.1007/s12032-016-0811-3) contains supplementary material, which is available to authorized users.

## Introduction

Approximately, 20–40 % of all patients with non-small cell lung cancer (NSCLC) present with brain metastasis (BM) at the time of diagnosis or develop BM during their disease course [1, 2]. The incidence of BM in patients with epidermal growth factor receptor (EGFR) mutant advanced NSCLC is higher than in patients with wild type EGFR over the disease course [3]. Moreover, the longer survival achieved with effective treatments such as EGFR tyrosine kinase inhibitors (TKIs) in EGFR mutant NSCLC is associated with a higher incidence of brain metastasis during the disease course.

Whole brain radiation therapy (WBRT) has been considered the standard treatment for BM, but usually results in neurologic sequelae such as neurocognitive dysfunction. Stereotactic radiosurgery (SRS) is a novel technique that is usually indicated in patients with oligo-brain metastasis. This technique reduces the radiation damage to the surrounding normal brain tissue, thereby resulting in less neurologic toxicity. Previous studies have demonstrated that supplementation of WBRT with SRS treatment does not confer any overall survival (OS) benefit compared with WBRT alone [4, 5]. In general, the prognosis of patients with BM in NSCLC remains poor (median survival time 3–6 months), even when active treatments such as WBRT and SRS are given [6, 7].

Achieving a detailed understanding of the molecular pathways of lung cancer has improved the clinical outcomes of patients with NSCLC [8]. Patients with NSCLC who harbor mutations in the epidermal growth factor receptor (EGFR) have been shown to exhibit high sensitivity to EGFR tyrosine kinase inhibitors (TKIs) [9, 10]. A number of recent large randomized phase III trials compared EGFR TKIs such as gefitinib, erlotinib, and afatinib with systemic chemotherapy. These trials consistently demonstrated higher response rates and longer progression-free survival (PFS) with EGFR TKI treatment in patients with EGFR mutant NSCLC, which resulted in EGFR TKIs being used as the standard first-line therapy [11–13].

Although the brain-to-plasma concentration ratios of EGFR TKIs are low (<1–3 %), several prospective studies have demonstrated that EGFR TKIs show promising activity, with a response rate of up to 80 % in patients with EGFR mutant NSCLC and brain metastasis [14, 15]. Nevertheless, WBRT and SRS are still commonly used to treat patients with BM before EGFR TKIs are used, even in patients with asymptomatic brain metastasis. In this context, the potential contribution of sequential cranial radiotherapy in patients with EGFR mutant NSCLC who are treated with EGFR TKIs remains to be determined.

Here, we performed a retrospective analysis to compare the clinical outcomes of patients treated first with cranial radiotherapy (WBRT or SRS) and then with EGFR TKIs with the outcomes of patients treated with EGFR TKIs alone.

## Patients and methods

### Patients

Clinical data were obtained by reviewing all pertinent medical records from a database at Samsung Medical Center. Between February 2005 to December 2013, data from patients who had a confirmed EGFR mutation (exon 19 deletion or the L858R point mutation), histologically proven NSCLC, clinical stage IIIB/IV or recurrent cancer with brain metastasis at the time of initial diagnosis, Eastern Cooperative Oncology Group performance status 0–2 and who were treated with EGFR TKIs (gefitinib or erlotinib) as the first-line therapy were retrospectively reviewed. Baseline patient characteristics collected for analysis included age, sex, smoking history, ECOG performance status, stage, number of metastasis sites, CNS symptoms, type of EGFR mutation, and type of EGFR TKI. Patients were treated with the recommended dose of either gefitinib (250 mg per day; oral delivery) or erlotinib (150 mg per day; oral delivery) until disease progression or unacceptable toxicity was documented. Responses were evaluated every 8 weeks with chest CT, while the patients were on therapy. Brain MRI was repeated every 3 months in patients who received SRS or WBRT, whereas it was performed only when indicated for patients treated with EGFR TKIs alone. Institutional review board approval was obtained from Samsung Medical Center (SMC; Seoul, Korea, 2016-02-005).

### Cranial radiotherapy

Patients were classified into two groups. Group A consisted of patients treated with cranial radiotherapy (WBRT or SRS) followed by EGFR TKIs, whereas group B consisted of patients treated with EGFR TKIs alone. WBRT was delivered using megavoltage machines with photon beams ranging from 4 to 10 MV through parallel opposed or 5 degree RAO–LAO fields that covered the entire cranial content. The eyes were excluded from the beam by either field arrangement or shielding. A dose of 2000 cGy was given daily for 5 days over a single week, yielding a total dose of 2000 cGy. SRS treatment involves a single high dose of stereotactically focused radiation. Gamma knife surgery consists of SRS using g-rays from radioactive cobalt-60 installed in a Gamma Knife system (Eleka Instruments, Stockholm, Sweden). EGFR TKI use was discontinued during SRS and WBRT.

### Statistical analysis

All available retrospective data were collected on a standardized data collection form. The objective of the present study was to compare the clinical outcomes of patients treated with cranial radiotherapy followed by EGFR TKIs with those of patients treated with EGFR TKIs alone. The primary outcome variable was OS. The secondary outcome variables included intracranial and extracranial PFS. OS was calculated from the start of EGFR TKI therapy until death or the last follow-up. PFS was calculated from the start of EGFR TKI therapy until disease progression, death without documented progression, or the last follow-up. Time to progression and survival were calculated using the Kaplan–Meier method. The log-rank test was used to test the significance of differences between the two groups. A Cox proportional hazards regression model was used to identify independent factors associated with OS or PFS. Two-sided *p* values <0.05 were considered to indicate significance. All analyses were performed using SPSS ver. 22.0 (IBM Corporation) software.

## Results

### Patient characteristics

Between January 2005 and December 2013, 573 patients at Samsung Medical Center who harbored an EGFR mutation received an EGFR TKI for NSCLC with brain metastasis. Among them, 121 patients (21.1 %) had brain metastasis at the time of initial diagnosis. The median patient age was 60 years (range 30–86 years), and 69 % of the patients were female. A total of 93 patients (77 %) were never smokers, and 98 patients (81 %) had extracranial metastasis at the time of diagnosis. The most common extracranial metastasis site was bone (56 %). Patients were treated with gefitinib (*n* = 103) or erlotinib (*n* = 18) as the first-line therapy. Group A consisted of 59 patients who were treated with additive therapy (32 with SRS, 26 with WBRT, and 1 patient with both), whereas group B consisted of 62 patients who were treated only with an EGFR TKI. Brain metastasis-related symptoms were observed in 28 patients (47 %) in group A, whereas they were observed in 12 patients (19 %) in group B. The number of patients with ≥5 BMs was higher in group B (76 %) than in group A (59 %). Twenty patients (17 %) had leptomeningeal carcinomatosis that was confirmed by cerebrospinal fluid cytology analysis (Table 1).

**Table 1:** Patient baseline characteristics (*N* = 121)

	Group A (*n* = 59) *n* (%)	Group B (*n* = 62) *n* (%)	Total (*n* = 121) *n* (%)
Sex			
Male	23 (39)	15 (24)	38 (31)
Female	36 (61)	47 (76)	83 (69)
Age (years)			
Median (range)	60 (30–86)	60 (33–80)	60 (30–86)
<65	43 (73)	37 (60)	80
≥65	16 (27)	25 (4)	41
Smoking status			
Never	45 (76)	48 (78)	93 (77)
Prior	10 (17)	12 (19)	22 (18)
Current	4 (7)	2 (3)	6 (5)
ECOG PS			
0	5 (9)	4 (7)	9 (7)
1	36 (61)	43 (69)	79 (65)
2	18 (30)	15 (24)	33 (28)
EGFR mutation			
Exon 19 deletion	39 (66)	35 (57)	74 (61)
Exon 21 L858R	20 (34)	27 (43)	47 (39)
EGFR TKI			
Gefitinib	54 (91)	49 (79)	103 (85)
Erlotinib	5 (9)	13 (21)	18 (15)
Extracranial metastasis			
None	19 (32)	4 (7)	23 (19)
One	18 (31)	28 (45)	46 (38)
≥Two	22 (37)	30 (48)	52 (43)
Site of extracranial metastasis			
Bone	28 (48)	40 (65)	68 (56)
Lung	17 (29)	22 (36)	39 (32)
Liver	10 (17)	10 (16)	20 (17)
Pleura	6 (10)	17 (27)	23 (19)
Adrenal gland	9 (15)	2 (3)	11 (9)
Other	4 (7)	5 (8)	9 (7)
Number of BMs			
<5	21 (36)	15 (24)	36 (30)
≥5	38 (64)	47 (76)	85 (70)
Co-existing LMS			
Yes	10 (17)	10 (16)	20 (17)
No	49 (83)	52 (84)	101 (93)
BM-related symptoms			
Yes	28 (48)	12 (20)	40 (33)
No	31 (52)	50 (80)	81 (67)

*ECOG PS* Eastern Cooperative Oncology Group performance status, *EGFR* epidermal growth factor receptor, *TKI* tyrosine kinase inhibitor, *BM* brain metastasis, *LMS* leptomeningeal carcinomatosis

### Treatment outcomes

**Fig. 1:**
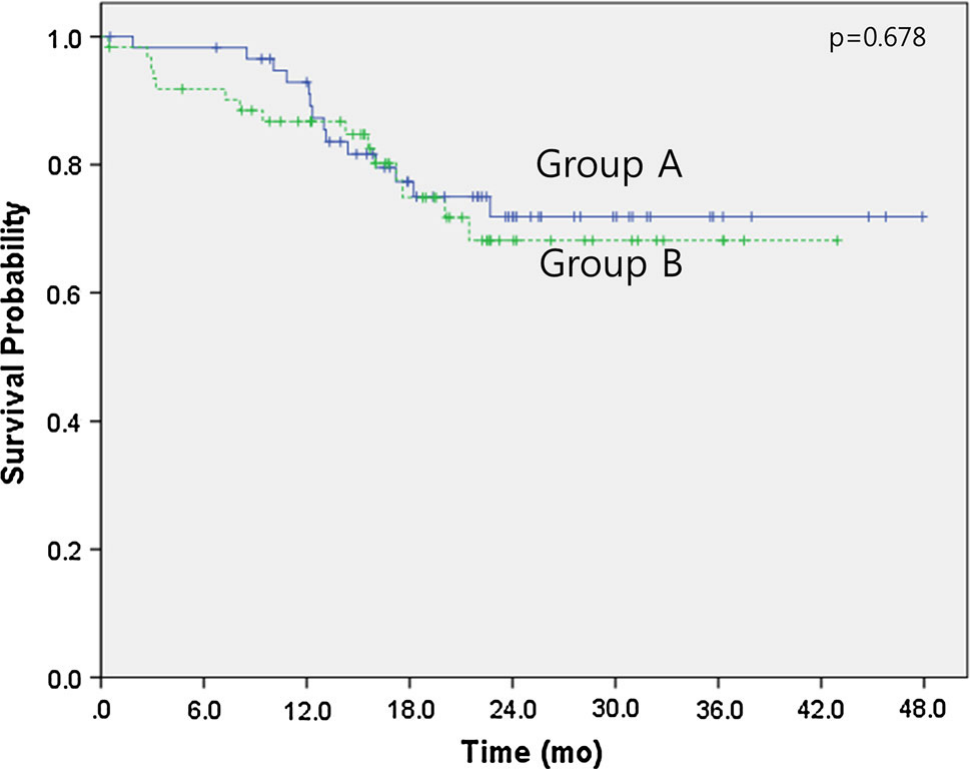
Kaplan–Meier survival curves. Overall survival of group A (cranial radiotherapy plus EGFR TKI) and group B (EGFR TKI only)

**Fig. 2:**
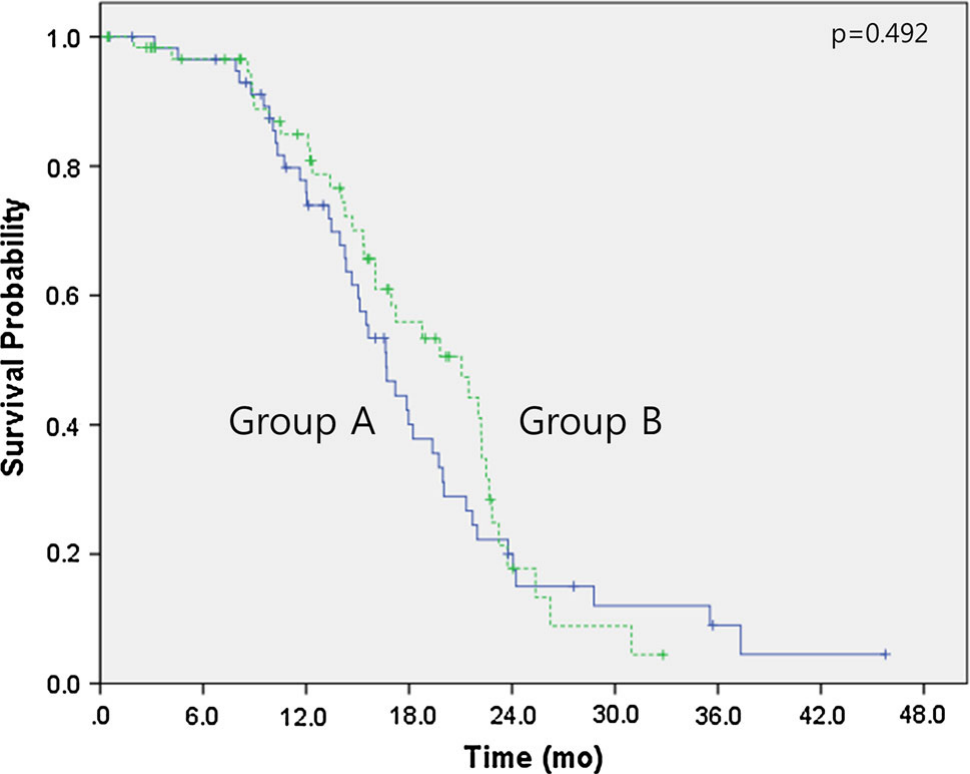
Kaplan–Meier survival curves. Intracranial progression-free survival of group A (cranial radiotherapy plus EGFR TKI) and group B (EGFR TKI only)

**Fig. 3:**
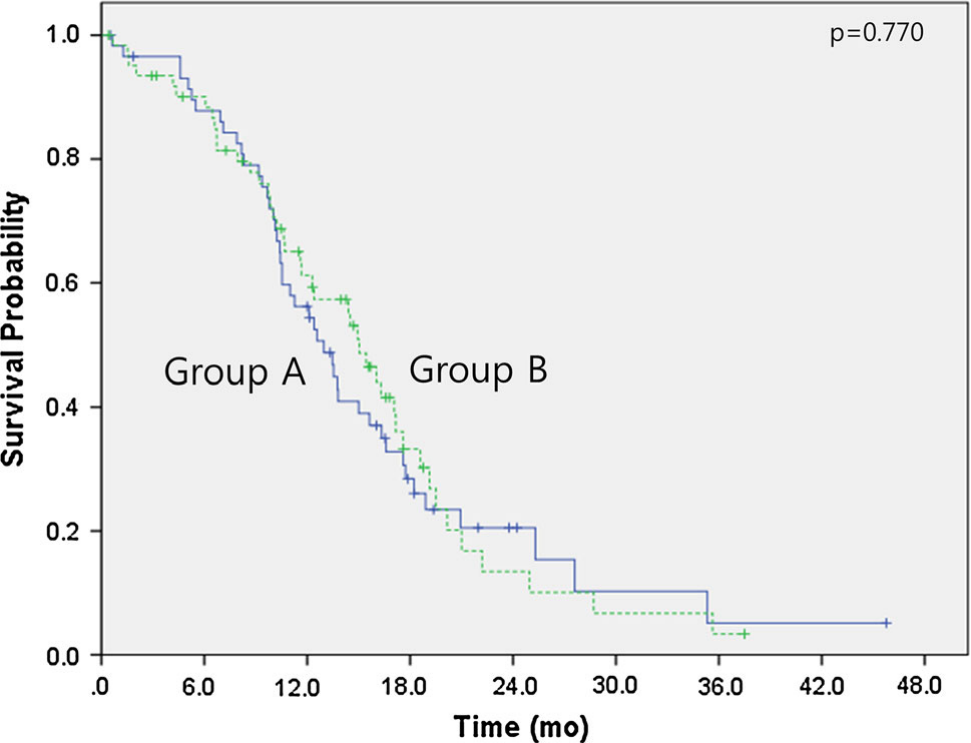
Kaplan–Meier survival curves. Extracranial progression-free survival of group A (cranial radiotherapy plus EGFR TKI) and group B (EGFR TKI only). *SRS* stereotactic surgery, *WBRT* whole brain radiation therapy

The median follow-up duration time was 18.4 months (range 0.4–47.9 months). Over this time, the median overall survival had not been reached in either group by the cutoff day (May 1, 2015). The estimated 3-year survival rates were 71.9 % for group A and 68.2 % for group B (*p* = 0.678) (Fig. 1). No significant differences were observed between the two groups regarding intracranial PFS (16.6 months in group A vs 21.0 months in group B, *p* = 0.492) (Fig. 2) or extracranial PFS (12.9 months in group A vs 15.0 months in group B, *p* = 0.770) (Fig. 3). However, the intracranial disease control rate (sum of complete response, partial response, and stable disease) was higher in group A compared with group B (79.7 vs 59.7 %, *p* = 0.019). No significant differences were observed between the two groups regarding the extracranial disease control rate (93.2 % in group A vs 93.5 % in group B, *p* = 0.942) or the objective response rate (sum of complete response and partial response) (83.1 % in group A vs 85.5 % in group B, *p* = 0.713). Salvage treatment of intracranial disease, including additional SRS or WBRT, was applied to a similar extent in both groups (11.9 % in group A vs 24.2 % in group B, *p* = 0.08).

Subgroup analysis of group A revealed that the median overall survival had not been reached in either group (SRS or WBRT). The estimated 3-year survival rate was 81.4 % for the SRS group and 62.2 % for the WBRT group (*p* = 0.106) (Supplementary Fig. S1). No significant difference was observed between the WBRT group and the SRS group regarding intracranial PFS (16.7 vs 15.6 months, *p* = 0.755) (Supplementary Fig. S2). Patients who were treated with SRS had longer extracranial PFS (16.3 vs 10.1 months, *p* = 0.008) compared with patients who were treated with WBRT (Supplementary Fig. S3). One patient who was treated with both SRS and WBRT was excluded from our analysis (*n* = 1).

### Prognostic factors

Multivariate analysis revealed that the number of BMs (≥5) [hazard ratio (HR) 3.36; 95 % CI 1.25–9.08, *p* = 0.016] and poor ECOG PS (2) (HR 3.66, 95 % CI 1.73–7.74, *p* = 0.001) were both independent factors for predicting poor OS. In addition, coexisting leptomeningeal carcinomatosis was an independent factor for predicting poor intracranial PFS (HR 1.79, 95 % CI 1.03–3.12, *p* = 0.04). Other variables such as sex, age (<65 vs ≥65 years old), specific EGFR TKI (gefitinib vs erlotinib), and extracranial metastasis (none vs present) did not influence survival outcome (Table 2).

**Table 2:** Multivariate analysis of prognostic factors for OS and PFS

	OS		Intracranial PFS		Extracranial PFS	
	HR (95 % CI)	*p*	HR (95 % CI)	*p*	HR (95 % CI)	*p*
Age (≥65)	1.72 (0.74–4.03)	NS	1.08 (0.60–1.97)	NS	0.85 (0.49–1.97)	NS
Sex (female)	1.61 (0.55–4.67)	NS	1.09 (0.66–4.67)	NS	0.78 (0.48–4.67)	NS
Smoking prior or current	1.09 (0.38–3.12)	NS	0.99 (0.54–1.80)	NS	1.62 (1.00–2.61)	NS
EGFR mutation (L858R)	0.42 (0.17–1.03)	NS	0.84 (0.52–1.36)	NS	0.98 (0.62–1.56)	NS
EGFR TKI (erlotinib)	0.40 (0.11–1.44)	NS	0.49 (0.22–1.44)	NS	0.83 (0.41–1.44)	NS
BM lesions (≥5)	3.36 (1.25–1.208)	0.016	1.65 (0.99–1.44)	NS	1.07 (0.63–1.44)	NS
Extracranial metastasis (yes)	1.32 (0.44–3.94)	NS	0.76 (0.38–1.50)	NS	0.90 (0.48–1.70)	NS
ECOG PS (2)	3.66 (1.73–7.74)	0.001	0.90 (0.36–1.70)	NS	1.58 (0.66–1.70)	NS
Co-existing LMS	0.78 (0.24 LMS70)	NS	1.79 (1.03–3.12)	0.04	1.12 (0.61–3.12)	NS

## Discussion

The brain is the one of the most common metastatic sites in lung cancer. The incidence of brain metastasis in patients with EGFR mutations is increasing due to the prolonged overall survival times achieved with effective targeting agents. Specifically, the use of EGFR TKIs extends survival times, thus allowing time for brain metastasis to develop. WBRT has been considered the standard treatment for patients with NSCLC and BM, even when the patients have asymptomatic or oligo-brain metastasis. However, long-term side effects such as neurocognitive dysfunction and memory loss often deter patients from receiving further anticancer therapy [16]. At present, SRS is widely used as an alternative treatment for oligo-brain metastasis. This treatment is less invasive and allows for precise tumor targeting, which minimizes the unintended irradiation of the adjacent normal tissue [17, 18].

The results of large randomized trials have indicated EGFR TKI treatment as the first-line therapy in patients with EGFR mutant NSCLC [19–21]. Importantly, EGFR TKIs can even cross the blood–brain barrier and have been shown to accumulate in brain metastatic lesions [22]. These compounds have also been shown to improve OS and intracranial PFS in patients with EGFR mutant NSCLC [6, 7, 23]. Given the inconsistent results obtained with the concurrent use of EGFR TKIs and cranial radiotherapy [24–27], the optimal management of patients with EGFR mutant NSCLC with brain metastasis remains to be determined.

In the present study, 21 % of all patients presented with brain metastasis at the time of diagnosis, a finding that is consistent with previous results [28]. However, no significant difference was observed between patients treated with an EGFR TKI versus patients treated with a additive therapy of EGFR TKI treatment and cranial radiation regarding overall survival. The estimated 3-year OS rates were similar in both groups (71.9 % for group A and 68.2 % for group B). In addition, no significant differences were observed between the two groups regarding intracranial PFS or extracranial PFS, although the intracranial disease control rate was slightly higher in group A than in group B. The median intracranial PFS times in both groups were greater than 18 months, which is a quite promising result, considering that all of the patients had brain metastasis. Moreover, the finding that EGFR TKI treatment alone achieved a prolonged intracranial PFS of 21.0 months was quite remarkable, considering that the EGFR TKI alone group had more patients with a high number of brain metastases (≥5) compared with the additive therapy group (76 vs 64 %). We also found that patients treated with SRS had a higher estimated 3-year OS rate compared with patients treated with WBRT (81.4 vs 62.2 %). One possible explanation for this finding is that the number of patients with many brain metastases (using a cutoff of 5) was much lower in patients treated with SRS than in patients treated with WBRT (47 vs 88 %).

Although additive therapy resulted in a higher intracranial disease control rate (79.7 vs 59.7 %), additional salvage treatment was required for progressive intracranial lesions to a similar extent in both groups (11.9 % in group A vs 24.2 % in group B, *p* = 0.08). The optimal timing and modality of cranial radiotherapy thus depend on each patient’s symptoms and signs of intracranial disease.

A recent meta-analysis demonstrated that upfront cranial radiotherapy plus systemic chemotherapy improved survival outcomes compared with EGFR TKI treatment alone in patients with EGFR mutant NSCLC with BM [29]. However, this improvement was only noted in 2-year overall survival and not in intracranial PFS or extracranial PFS. Furthermore, limiting the analysis to six prospective studies revealed no significant differences in intracranial PFS or OS between cranial radiotherapy in additive with EGFR TKI treatment versus EGFR TKI treatment alone, which is consistent with the present study. Given the heterogeneous nature of the data obtained in the single arm studies included in the meta-analysis, the outcomes achieved with upfront cranial radiotherapy followed by EGFR TKI treatment should be compared in future randomized controlled studies with those achieved with EGFR TKI treatment alone in patients with EGFR mutant NSCLC and brain metastasis.

The main strength of this study is the homogeneity of the patient population. Specifically, all patients had an EGFR mutation, brain metastasis at the time of diagnosis, and underwent EGFR TKI therapy as the first-line treatment.

However, the present study also has some limitations. Given the retrospective nature of the analysis, undefined bias and/or confounding factors might have affected the clinical outcomes. For example, the decision to treat patients with WBRT, SRS, or EGFR TKIs was at the discretion of the treating physicians, which may have led to bias. Moreover, the follow-up intervals of brain imaging in the two groups were not equal, which might have affected our assessment of intracranial PFS. Finally, the sample size was relatively small, which implies that our results should be interpreted with caution.

In conclusion, additive therapy consisting of cranial radiotherapy followed by EGFR TKIs did not improve overall survival, intracranial PFS, or extracranial PFS compared with EGFR TKI treatment alone in patients with EGFR mutant NSCLC and brain metastasis. Further prospective randomized studies are needed to define the precise benefit of sequential cranial radiotherapy in this patient population.

## Electronic supplementary material

Below is the link to the electronic supplementary material.

Supplementary Fig. S1 Kaplan–Meier survival curves. Overall survival of patients treated with SRS or WBRT. (TIFF 446 kb)

Supplementary Fig. S2 Kaplan–Meier survival curves. Intracranial progression-free survival of patients treated with SRS or WBRT. (TIFF 511 kb)

Supplementary Fig. S3 Kaplan–Meier survival curves. Extracranial progression-free survival of patients treated with SRS or WBRT. (TIFF 478 kb)

Supplementary material 4 (DOCX 13 kb)
