# Supplementary figures and images for: Analysis of the benefit of sequential cranial radiotherapy in patients with EGFR mutant non-small cell lung cancer and brain metastasis
Source: Med Oncol. 2016 Jul 22;33:97. doi: 10.1007/s12032-016-0811-3 (PMC4958121; doi:10.1007/s12032-016-0811-3)

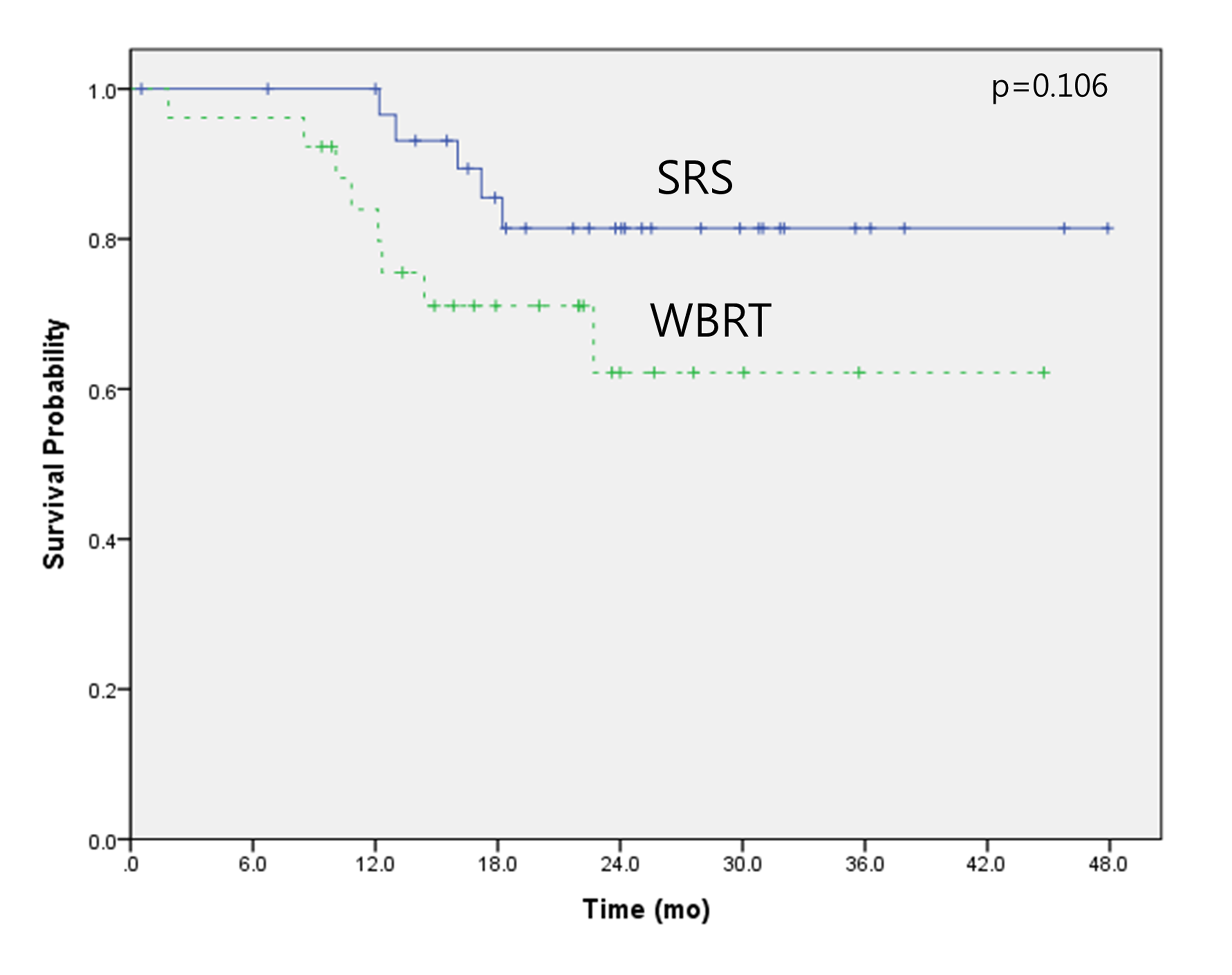

Supplement: Supplementary file 1 — Kaplan–Meier survival curves. Overall survival of patients treated with SRS or WBRT. (TIFF 446 kb) [file 12032_2016_811_MOESM1_ESM.tif]

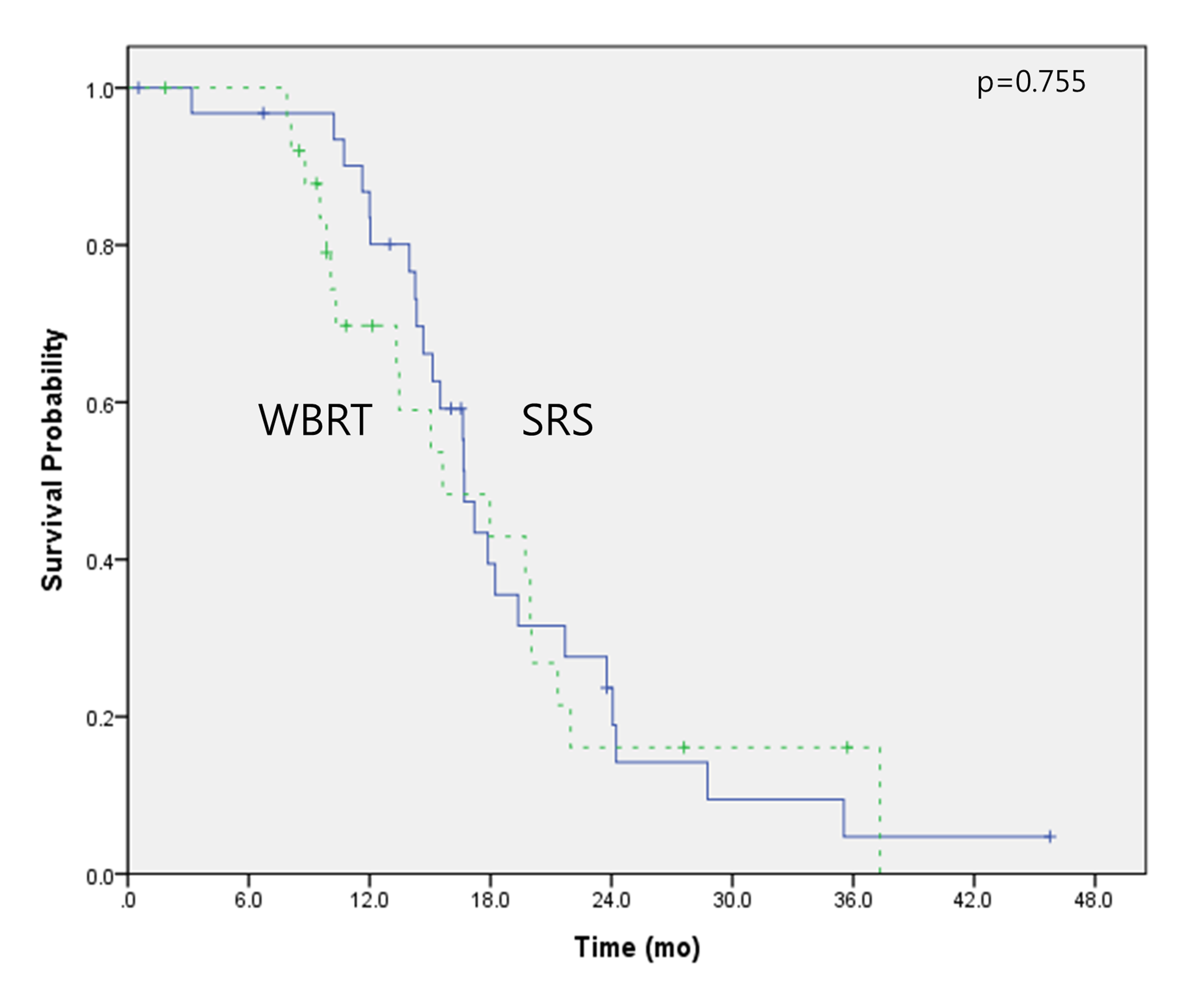

Supplement: Supplementary file 2 — Kaplan–Meier survival curves. Intracranial progression-free survival of patients treated with SRS or WBRT. (TIFF 511 kb) [file 12032_2016_811_MOESM2_ESM.tif]

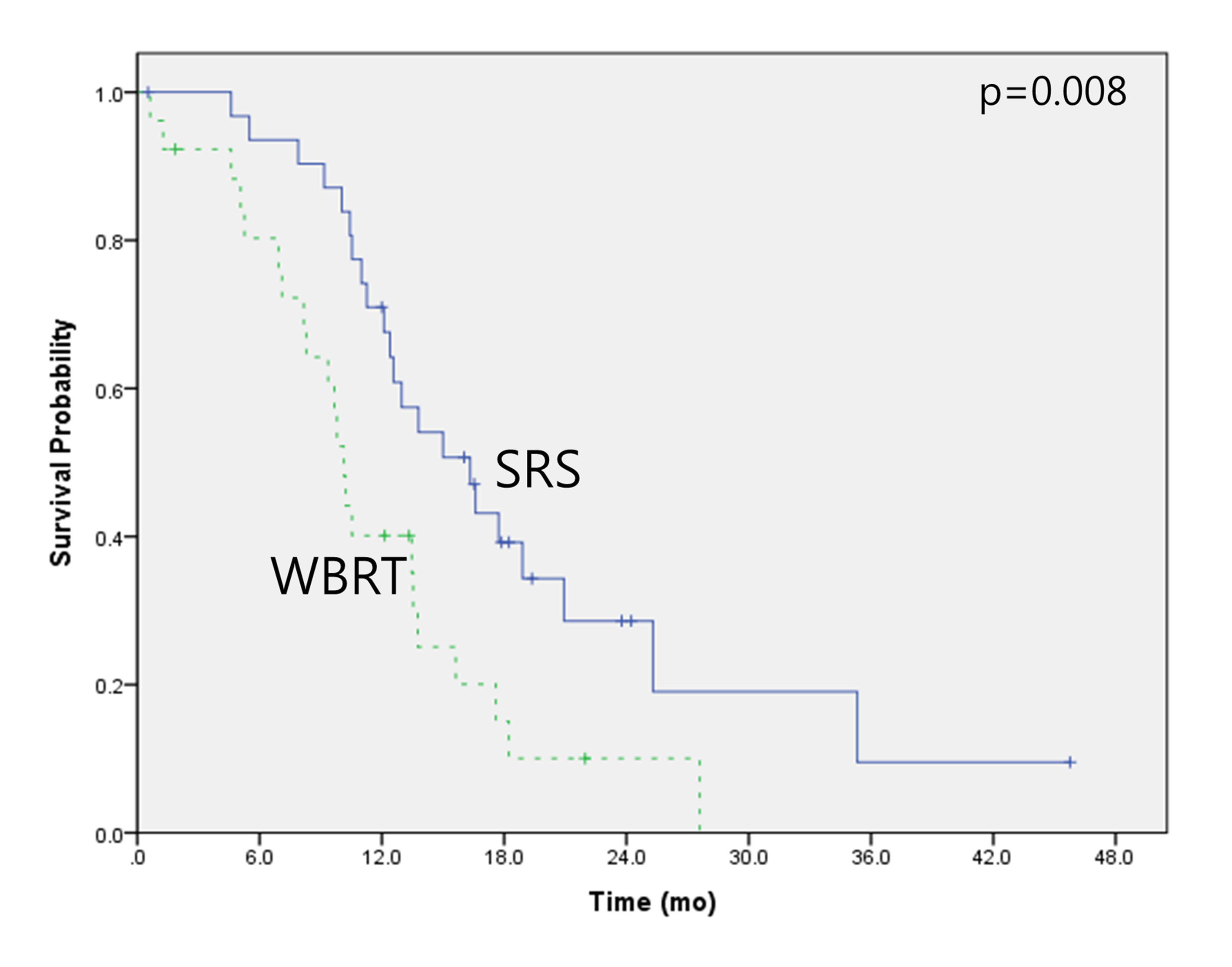

Supplement: Supplementary file 3 — Kaplan–Meier survival curves. Extracranial progression-free survival of patients treated with SRS or WBRT. (TIFF 478 kb) [file 12032_2016_811_MOESM3_ESM.tif]
