# Supplementary material for: Analysis of the benefit of sequential cranial radiotherapy in patients with EGFR mutant non-small cell lung cancer and brain metastasis
Source: Med Oncol. 2016 Jul 22;33:97. doi: 10.1007/s12032-016-0811-3 (PMC4958121; doi:10.1007/s12032-016-0811-3)
Supplement: Supplementary file 4 — Supplementary material 4 (DOCX 13 kb) [file 12032_2016_811_MOESM4_ESM.docx]

**Supplementary Table 1.** Subgroup analysis of patient baseline characteristics (N=58)

|  | SRS  (n=32)  n (%) | WBRT  (n=26)  n (%) |
| --- | --- | --- |
| Sex  Male  Female | 6 (19)  26 (81) | 16 (62)  10 (38) |
| Age  Median (range)  <65  >=65 | 56.5 (35 – 76)  22 (69)  10 (31) | 57 (30 – 74)  21 (81)  5 (19) |
| Smoking status  Never  Prior  Current | 28 (88)  3 (9)  1 (3) | 17 (65)  6 (23)  3 (12) |
| ECOG PS  0  1  2 | 4 (12)  20 (63)  8 (25) | 1 (4)  16 (61)  9 (35) |
| EGFR mutation  Exon 19 deletion  Exon 21 L858R | 21 (66)  11 (35) | 17 (65)  9 (35) |
| EGFR TKI  Gefitinib  Erlotinib | 29 (90)  3 (10) | 24 (92)  2 (8) |
| Extracranial metastasis  None  One  >= Two | 12 (38)  10 (31)  10 (31) | 7 (27)  8 (31)  11 (42) |
| Site of extracranial metastasis  Bone  Lung  Liver  Pleura  Adrenal gland  Other | 13 (40)  8 (25)  4 (12)  5 (16)  2 (6)  1 (3) | 14 (54)  8 (31)  5 (19)  1 (4)  7 (27)  3 (12) |
| Number of BMs  <5  >=5 | 17 (53  15 (47) | 3 (12)  23 (88) |
| Co-existing LMS  Yes  No | 3 (10)  29 (90) | 7 (27)  19 (73) |
| BM-related symptoms  Yes  No | 12 (38)  20 (62) | 15 (58)  11 (42) |
